# Autoimmune retinal microangiopathy

**DOI:** 10.22336/rjo.2026.66

**Published:** 2026

**Authors:** Angela Corduneanu, Natalia Corduneanu, Alina Moscalciuc

**Affiliations:** 1 Ophthalmology Department, „Timofei Moşneaga“ Republican Clinical Hospital, Chişinău, Republic of Moldova; 2 MedClinic International, Chişinau, Republic of Moldova

**Keywords:** dermatomyositis, proliferative vascular retinopathy, autoimmune connective tissue diseases, CTDs = Connective tissue diseases, DM = Dermatomyositis, BCVA = best corrected, SD-OCT = spectral domain optical coherence tomography, VEGF = vascular endothelial growth factor

## Abstract

**Purpose:**

To describe a patient with proliferative vascular retinopathy as an ocular complication of an autoimmune connective tissue disease.

**Method:**

Case report.

**Results:**

A 27-year-old female patient was referred for a regular eye examination, 5 months after the onset of dermatomyositis. On examination, visual acuity was 20/20 in both eyes. The anterior segment was normal. Funduscopy showed microhemorrhages and numerous retinal neovessels in both eyes. The established diagnosis was proliferative vascular retinopathy in dermatomyositis. The patient received treatment with multiple intravitreal anti-VEGF injections. Despite a good response to dermatomyositis treatment with methylprednisolone 4 mg per day and methotrexate 15 mg per week, the retinal neovascularisation reactivated within 3-4 months after anti-VEGF injections. Laser retinal photocoagulation has been performed, and the patient’s follow-up continues.

**Discussion:**

Proliferative vascular retinopathy represents an exceedingly rare ocular manifestation of dermatomyositis. The marked bilateral retinal capillary nonperfusion and recurrent neovascularization observed in this patient are consistent with a severe ischemic microangiopathic process, potentially related to immune-mediated endothelial injury. Only isolated cases of proliferative retinopathy associated with overlap connective tissue disease have been reported, underscoring the unusual nature of this case.

**Conclusion:**

Proliferative vascular retinopathy may occur secondary to dermatomyositis. All clinicians must recognize this rare association to detect and treat it early.

## Introduction

Connective tissue diseases (CTDs) comprise a heterogeneous group of autoimmune disorders characterized by chronic inflammation, immune-mediated vascular injury, and progressive tissue damage. Although systemic manifestations vary among diseases, microvascular dysfunction is increasingly recognized as a common pathogenic pathway underlying organ involvement in conditions such as dermatomyositis, systemic sclerosis, systemic lupus erythematosus, and mixed connective tissue disease. Endothelial injury, capillary rarefaction, vascular occlusion, and tissue ischemia contribute significantly to disease progression and clinical outcomes.

Dermatomyositis (DM) is a rare, idiopathic inflammatory autoimmune disease of muscle tissue characterized by proximal muscle weakness and a characteristic skin rash. The estimated incidence is 2-5 cases per million population. Females are affected twice as often as males. Heliotrope eyelid eruptions are considered a hallmark of DM affecting the eye. Other ocular manifestations of DM include conjunctival edema, nystagmus, extraocular muscle weakness, iritis, cotton wool spots, optic atrophy, and conjunctival pseudopolyposis. Retinopathy is also a rare presentation [1].

In this case report, we describe an unusual case of proliferative vascular retinopathy as an ocular complication of dermatomyositis.

## Case report

A 27-year-old Caucasian female with a diagnosis of dermatomyositis with esophageal muscle involvement, maintained on oral medication (Methypred 48 mg per day and Methotrexate 15 mg per week), presented with no vision complaints for a regular eye examination.

BCVA in each eye was 20/20. The anterior segment was unremarkable. On dilated exam, there were several dot hemorrhages and multiple new blood vessels (**Fig. 1A, B**). OCT of the macula showed abnormal macular thinning, which could indicate chronic retinal ischemia (**Fig. 2A, B**). Fluorescein angiography confirmed multiple retinal neovascularizations with late leakage in both eyes and massive capillary dropout (**Fig. 3A-F**). ** A B ** E \ F

**Fig. 1:**
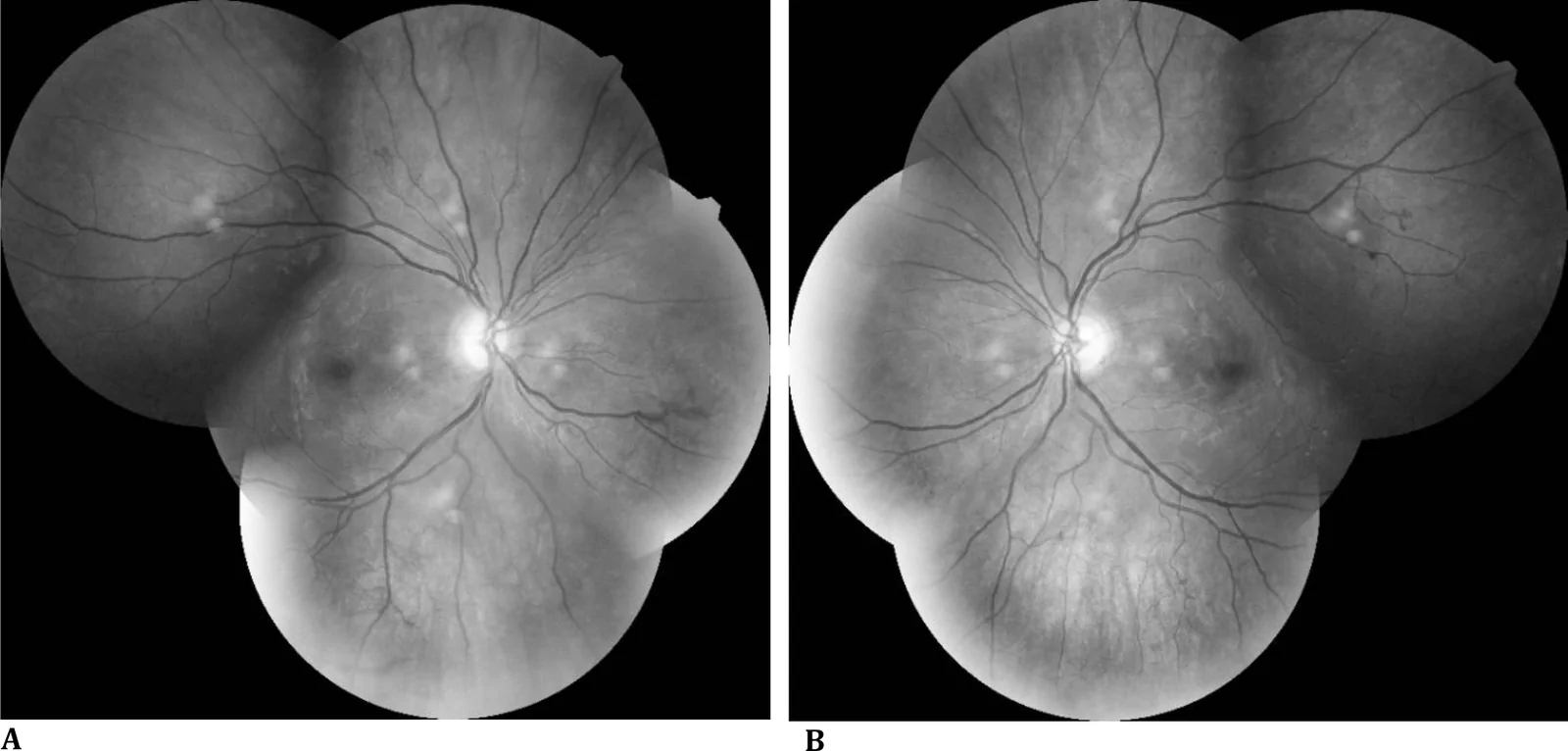
Montage of fundus photographs. (**A**) The right eye shows several dot hemorrhages, mostly in the macula, and multiple new vessels in the inferonasal and superior retina; (**B**) The left eye shows several dot hemorrhages, mostly in the macula, and new vessels in the superotemporal and inferior retina

**Fig. 2:**
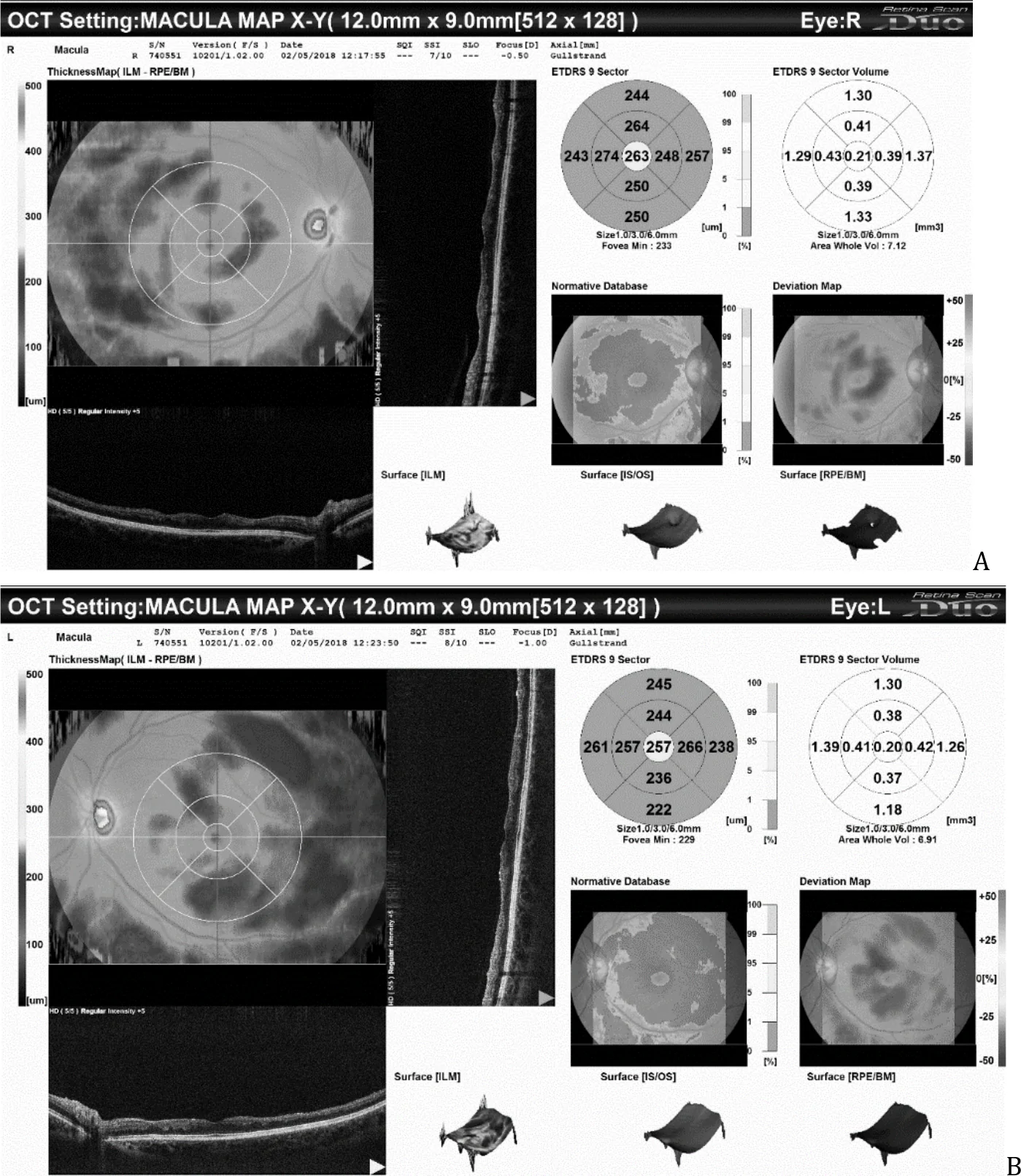
SD-OCT macula of (**A**) the right and (**B**) the left eye. The thickness map shows significant retinal thinning

**Fig. 3:**
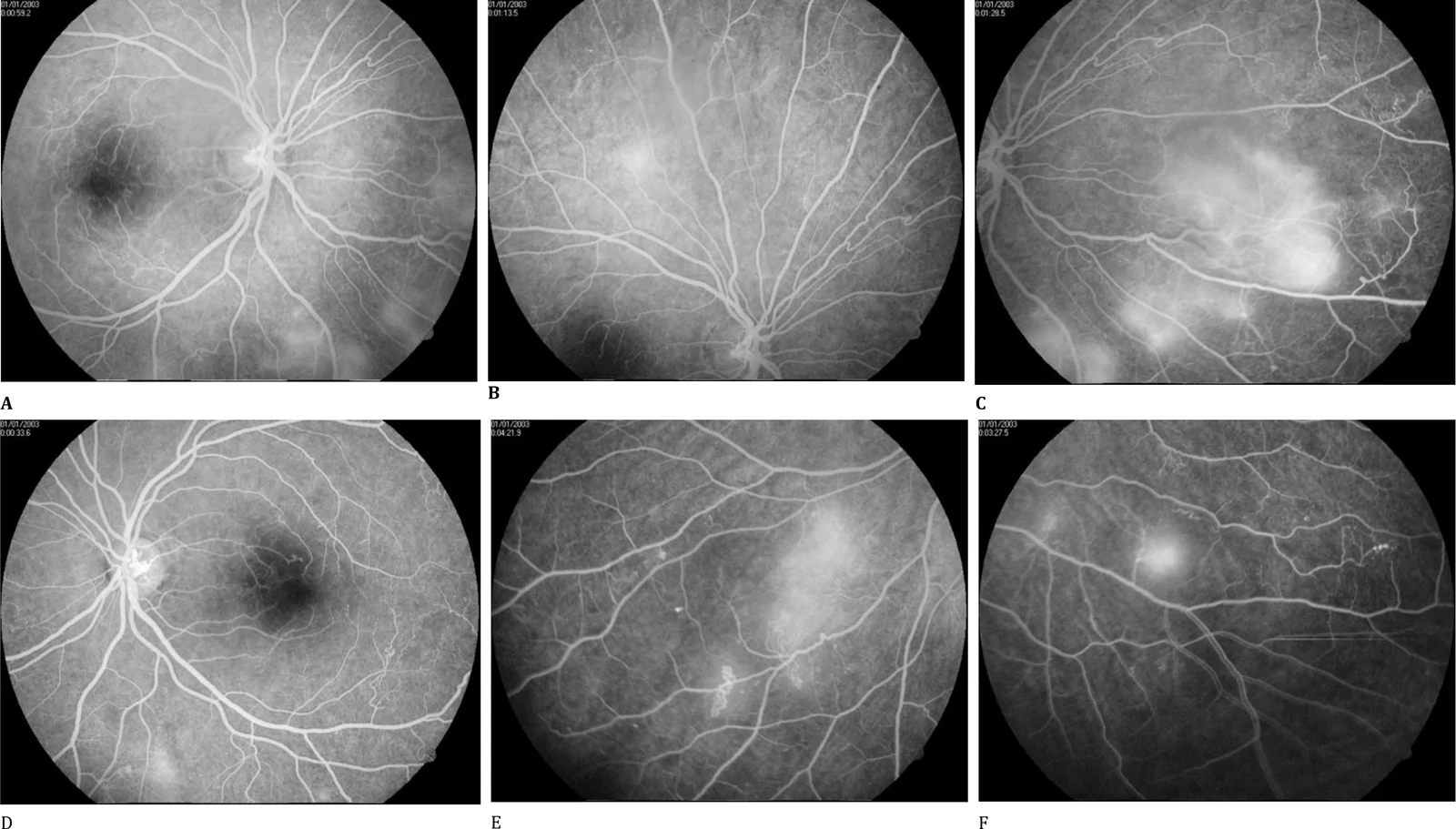
Late-phase fluorescein angiography images of the (**A-C**) right and (**D-F**) left eyes demonstrate retinal neovessels with late leakage

Therefore, the patient was diagnosed with proliferative vascular retinopathy and was started on treatment with intravitreal injections of anti-VEGF (Bevacizumab) in both eyes.

One month after intravitreal bevacizumab treatment, retinal neovascularization regressed (**Fig. 4A, B**).

**Fig. 4:**
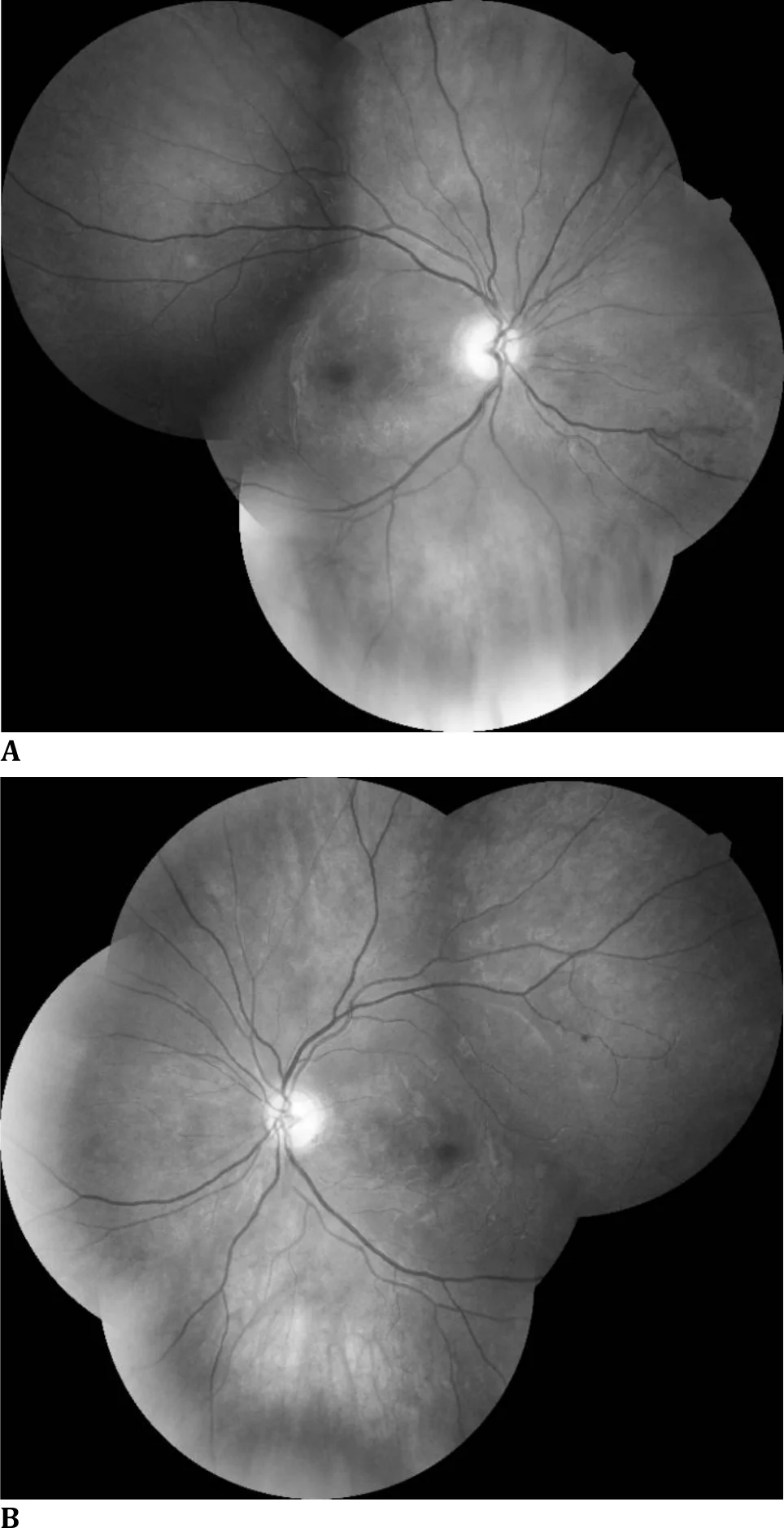
Montage fundus photograph of the (**A**) right and (**B**) left eye after 1 month of treatment, demonstrating the resolution of retinal neovascularization and retinal hemorrhages

However, after 3-4 months, retinal neovascularization recurred, so laser retinal photocoagulation was started, and intravitreal bevacizumab was continued every 3-4 months in both eyes.

Over the course of 2 years, the systemic treatment was gradually tapered to methylprednisolone 4 mg per day and methotrexate 15 mg per week. Over this period, the patient received 4 intravitreal injections with bevacizumab in each eye because of retinal neovascularization relapse.

From 2018 until the last eye examination in February 2020, no neovascularization was detected, and no ocular treatment was needed (**Fig. 5**).

**Fig. 5:**
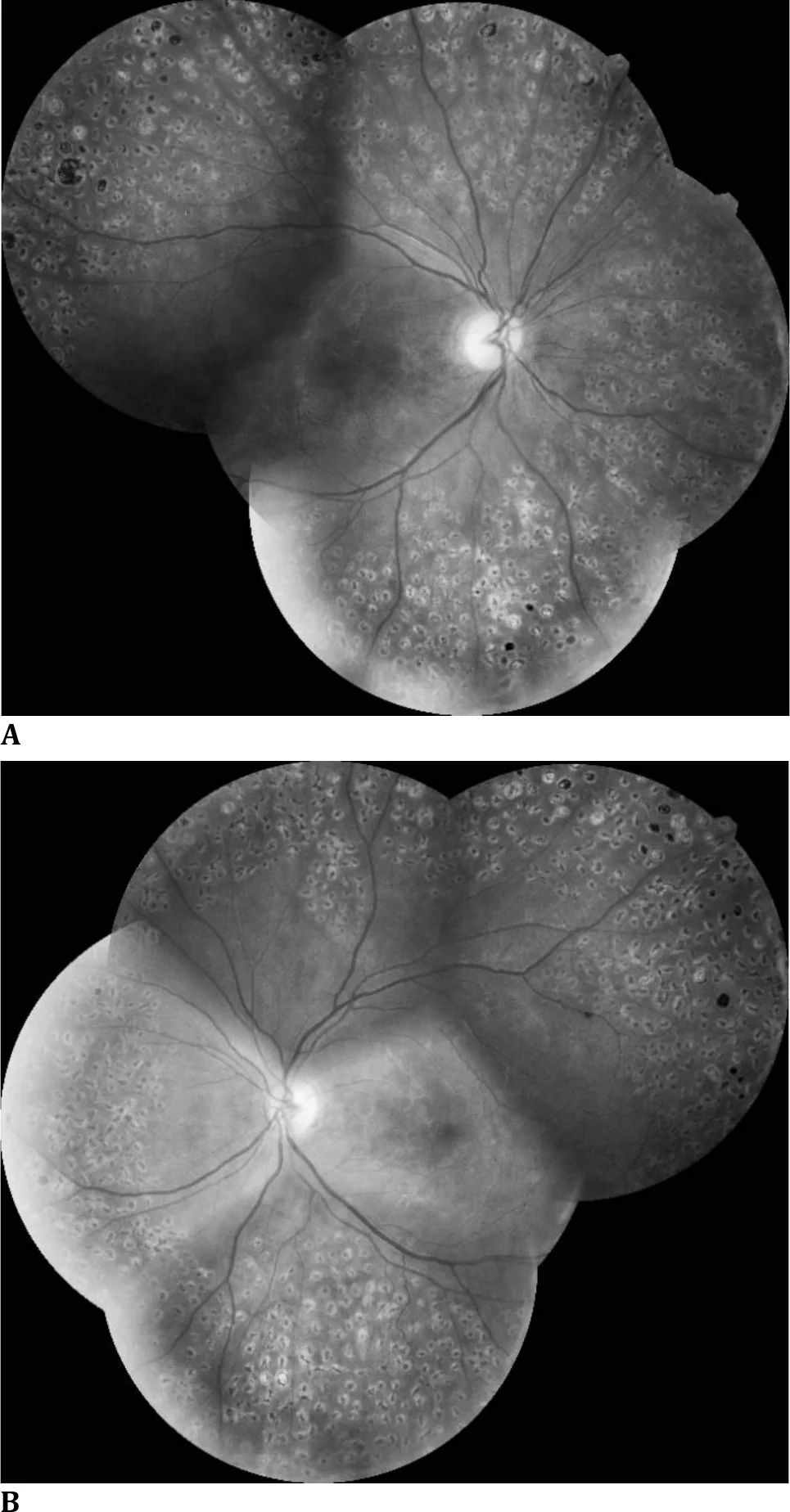
Montage fundus photograph of the (**A**) right and (**B**) left eye after 4 years of treatment. Areas of panretinal photocoagulation and no new blood vessels or retinal hemorrhages are observed

On the last eye examination, BCVA in each eye was 18/20 due to posterior subcapsular cataract.

## Discussion

Our patient with dermatomyositis, without other systemic or ocular risk factors, presented with proliferative retinopathy in both eyes, had no visual complaints, and was managed with intravitreal injections of Bevacizumab and panretinal laser photocoagulation in both eyes.

The posterior segment findings associated with dermatomyositis are hypothesized to result from immune-complex–mediated vasculitis that destroys small capillary beds [2].

Although the precise mechanisms of dermatomyositis are unknown, earlier models and more recent genomic analyses of skin and muscle samples suggest involvement of complement-mediated microangiopathy and exposure to type 1 interferons, specifically interferon-β [3].

In 2007, Venkatesh P et al. described 2 cases of proliferative vascular retinopathy in two patients with polymyositis/dermatomyositis with scleroderma (overlap syndrome), characterized by hemiretinal and branch retinal vascular occlusion with associated capillary nonperfusion areas and retinal and disc neovascularization [4].

Although retinopathy may be underdiagnosed in patients with CTDs, it remains asymptomatic until vision-threatening complications occur.

Retinopathy in dermatomyositis is rare, and few reports note visual loss from macular hemorrhage or macular edema [5].

## Conclusion

This case highlights the importance of funduscopic examination in patients with dermatomyositis, even without ocular symptoms, because the macula may be spared early in the process. Prompt recognition of peripheral retinal complications and appropriate treatment could prevent subsequent ocular complications.

## Presentation at a conference

This case was presented as an e-poster at Euretina 2021 Virtual: https://euretina.org/resource/abstract_2021_bilateral-proliferative-vascular-retinopathy-as-a-manifestation-of-dermatomyositis-case-report/.
